# Occupational Performance Coaching for Children With Neurodisability: A Randomized Controlled Trial Protocol

**DOI:** 10.1177/00084174231160976

**Published:** 2023-03-15

**Authors:** Fiona P. Graham, Jonathan A. Williman, Laura N. Desha, Deborah Snell, Bernadette Jones, Tristram R. Ingham, Anna Latu, Jasjot K. Maggo, Annemarei Ranta, Jenny Ziviani

**Keywords:** Neurodisability, Translational research, Child development, International classification of functioning-disability and health, Self-determination, Autodétermination, classification internationale du fonctionnement, du handicap et de la santé, développement de l’enfant, handicaps neurologiques, recherche translationnelle

## Abstract

**Background.** Occupational Performance Coaching (OPC) is a goal-oriented approach in which client agency takes precedence in goal selection, analysis, choice of action, and evaluation of success. The intended outcomes of OPC are improved occupational performance and participation in clients’ life situations. Randomized clinical trials are needed to determine the effectiveness of OPC. **Purpose.** This study protocol outlines a randomized controlled trial (RCT) of OPC compared to usual care with caregivers of children with neurodisability in improving child, caregiver, and family occupational performance. **Method.** A single-blind, 2-arm parallel-group, cluster RCT of OPC compared to usual care is planned. Therapists delivering the intervention (N = 14) are randomized to “OPC training” or “usual care” groups. The primary outcome is occupational performance improvement in caregiver (N = 84) identified goals. **Implications.** Findings will provide translational evidence of the effectiveness of OPC and clarify intervention processes. Areas of future OPC research and development will be indicated.

## Résumé

**Description.** Le coaching en rendement occupationnel (CRO) est une approche axée sur les objectifs à atteindre dans laquelle l’agentivité de la clientèle joue un rôle prédominant dans la sélection des objectifs, l’analyse, le choix des actions et l’évaluation du succès. Les résultats attendus du CRO sont l’amélioration du rendement occupationnel et de la participation aux activités de la vie de la personne. Des essais cliniques randomisés sont nécessaires pour déterminer l’efficacité du CRO. **But.** Ce protocole de recherche décrit un essai clinique randomisé réalisé auprès des aidants d’enfants ayant une expérience du handicap neurologique et comparant les résultats du CRO avec ceux des soins habituels pour l’amélioration du rendement occupationnel de l’enfant, du proche aidant et de la famille. **Méthodologie.** Un essai clinique randomisé en grappe, en simple aveugle et à deux groupes parallèles comparant le CRO aux soins habituels est prévu. Les thérapeutes chargés de l’intervention (N = 14) sont répartis aléatoirement dans les groupes « formation CRO » ou « soins habituels ». Le résultat primaire est l’amélioration du rendement occupationnel dans les objectifs identifiés par les aidants (N = 84). **Conséquences.** Les résultats fourniront des preuves translationnelles de l’efficacité du CRO et clarifieront les processus d’intervention. Les domaines de recherche et de développement futurs du CRO seront indiqués.

## Background

Neuro-developmental disability (ND) is an umbrella term describing a range of health conditions (e.g., Autism Spectrum Disorder, Cerebral Palsy, Attention Deficit, and Hyperactivity Disorder) that involve impairment to the developing nervous systems and contribute to functional limitations with onset during childhood (Morris et al., 2013). Disability-related impacts of ND can involve a persons’ physical, cognitive, social, and communication abilities (Morris et al., 2013). Participation in life situations that are valued by children with ND and their families is a key outcome of occupational therapy (Layton, 2017; Restall & Egan, 2021) and the primary intended outcome of Occupational Performance Coaching (OPC: Graham & Ziviani, 2021b).

‘Participation’ involves a person's capacity (e.g., physical or communicative ability), their subjective experience, and the affordance of the environment to support or restrict participation (Imms et al., 2016) Occupational performance and participation are closely related concepts that share a focus on personally valued enactment of societal roles (Graham & Ziviani, 2021b; Imms, 2006). Children with ND are more likely to experience restricted frequency and diversity of participation in comparison with their peers (Imms et al., 2008; Orsmond et al., 2013), discrimination that prevents participation (Huus et al., 2021) and to demonstrate a pattern of decline in participation over time (Majnemer et al., 2015). Interventions are therefore needed that improve the quality of participation experiences for children with ND by addressing the specific abilities and perspectives of the child and family, and the environmental influences on participation. Coaching is proposed as an intervention that can attend to the complex conditions (Novak, 2014) and an alternative to the predominant impairment-oriented interventions in paediatric rehabilitation (Anaby et al., 2017).

A partnership approach is used in coaching to guide clients’ realization of personal priorities (Pentland & Heinz, 2016). In rehabilitation contexts, coaching prompts a shift in focus from what therapists know about clients to how therapists relate to clients in order to amplify client agency. Although there is variation between specific coaching interventions (Kessler & Graham, 2015; Schwellnus et al., 2015), common components include collaborative exploration of future-oriented goals, with an emphasis on client-led reflection, observation, and evaluation of progress (Rush & Sheldon, 2011; Schwellnus et al., 2015).

Coaching of caregivers of children with ND is emerging as a key feature of contemporary evidence-based interventions intending to influence child outcomes (Akhbari Ziegler et al., 2019; Landa, 2018; Missiuna et al., 2012; Novak et al., 2020; Oono et al., 2013). OPC is theoretically grounded in family centred care (Rosenbaum, 1998), the person–environment–occupation model (Law et al., 1996), self-determination theory (Deci & Ryan, 1985), and adult learning theory (Knowles et al., 2005). The family (often in effect the parent/caregiver–child dyad) is the “client.” Client needs for autonomy, relatedness, and competence (Deci & Ryan, 1985) are responded to within the development of a client-directed plan. Clients’ pre-existing knowledge and resources (Knowles et al., 2005) are highlighted and linked to discussions of ways to progress toward goals. Specific OPC components (Collaborative Performance Analysis) guide therapists in prioritizing client knowledge, observation, and reflection over their own expertise, such as knowledge of health conditions and health systems. A logic model for OPC is available (https://www.otago.ac.nz/opc/resources). International (Anaby et al., 2017) and in Aotearoa/New Zealand (Graham et al., 2020; Widdowson et al., 2015) evidence indicates, that while usual care for children with ND is variable, it predominantly reflects directive communication by therapists, infrequent use of goal setting and targeting of children's impairments, thus is substantially different to OPC.

Proof-of-concept that OPC positively influences child, parent, and teacher outcomes has been established in studies conducted in India (N = 36, Angelin et al., 2021), Australia (N = 16, Bernie et al., in press; N = 3, Graham et al., 2010; N = 29, Graham et al., 2013); Hong Kong (N = 4, Chien et al., 2020); Canada (N = 11, Hui et al., 2016) and Germany (N = 3, Kennedy-Behr et al., 2013). Initial randomized controlled trials (RCTs) of OPC with caregivers of children with ND found significant improvement in individualized participation-oriented goals for children with cerebral palsy (N = 43, Jamali et al., 2022; N = 30, Kahjoogh et al., 2019). Both prior RCTs of OPC reflect efficacy study designs in which a single therapist applied OPC within research-oriented recruitment systems. Neither RCT reported intervention fidelity processes that assure that OPC was implemented as intended (Graham & Ziviani, 2021a). While important early steps in the development of evidence, translational research (Woolf, 2008) on OPC is now needed.

The cultural fit of OPC with Māori, the indigenous people of Aotearoa/New Zealand, warrants exploration. Māori are over-represented in many health conditions leading to ND, for example, cerebral palsy and traumatic brain injury. The needs of Māori may be better meet by interventions like OPC that enhance whānau (family) mana (personal authority, status) by promoting active involvement of family, and specific attention to relationship building (whakawhanaungatanga) (Bevan-Brown, 2004; Harwood et al., 2012; Wong et al., 2021). However, Māori caregivers’ experiences of OPC and the need for tailoring of OPC for Māori remain unknown.

### Study Objectives and Hypotheses

The primary objective of this study is to determine if children with ND and their parents /primary caregivers^1^ (i.e., child–caregiver dyads) who receive OPC in service delivery contexts, experience improved participation in personally selected life situations compared to usual care. We hypothesise that compared with dyads receiving usual care, dyads receiving OPC will demonstrate greater participation in personally valued life situations as measured by the Canadian Occupational Performance Measure (COPM) (Law et al., 2005).

The secondary objectives of the study are to examine the extent to which implementation of OPC impacts children's functional ability and quality of life, and caregiver mental health; determining if OPC is cost effective compared to usual care; and if outcomes vary according to intervention fidelity and dose. We hypothesise that dyads receiving OPC will demonstrate: (a) greater improvement in child functional ability as measured by the Pediatric Evaluation of Disability Inventory (PEDI-CAT) (Haley et al., 2011), (b) greater quality of life as measured by the KIDSCREEN10, and (c) more positive caregiver mental health as measured by the Depression Anxiety Stress Scale (DASS) (Antony et al., 1998). Previous qualitative (Graham et al., 2010) and quantitative (Chien et al., 2020; Graham et al., 2013) research on OPC have indicated positive effects of OPC on maternal mental health thus indicating this as an important outcome variable in a fully powered analysis of OPC effectiveness. We also hypothesise that OPC intervention is more cost-effective based on COPM outcomes and therapy dosage (session number, length, and frequency).

A further objective is to explore the perspectives of Māori caregivers on the experience of receiving OPC, shared through qualitative interviews. Group differences for Māori in the primary outcome (COPM) will also be analyzed to determine treatment effects for Māori.

### Ethics

Ethical approval has been obtained from [removed for blind peer review] each employer locality and their corresponding Māori consultation committees.

## Method

### Trial Design

**Figure 1.**
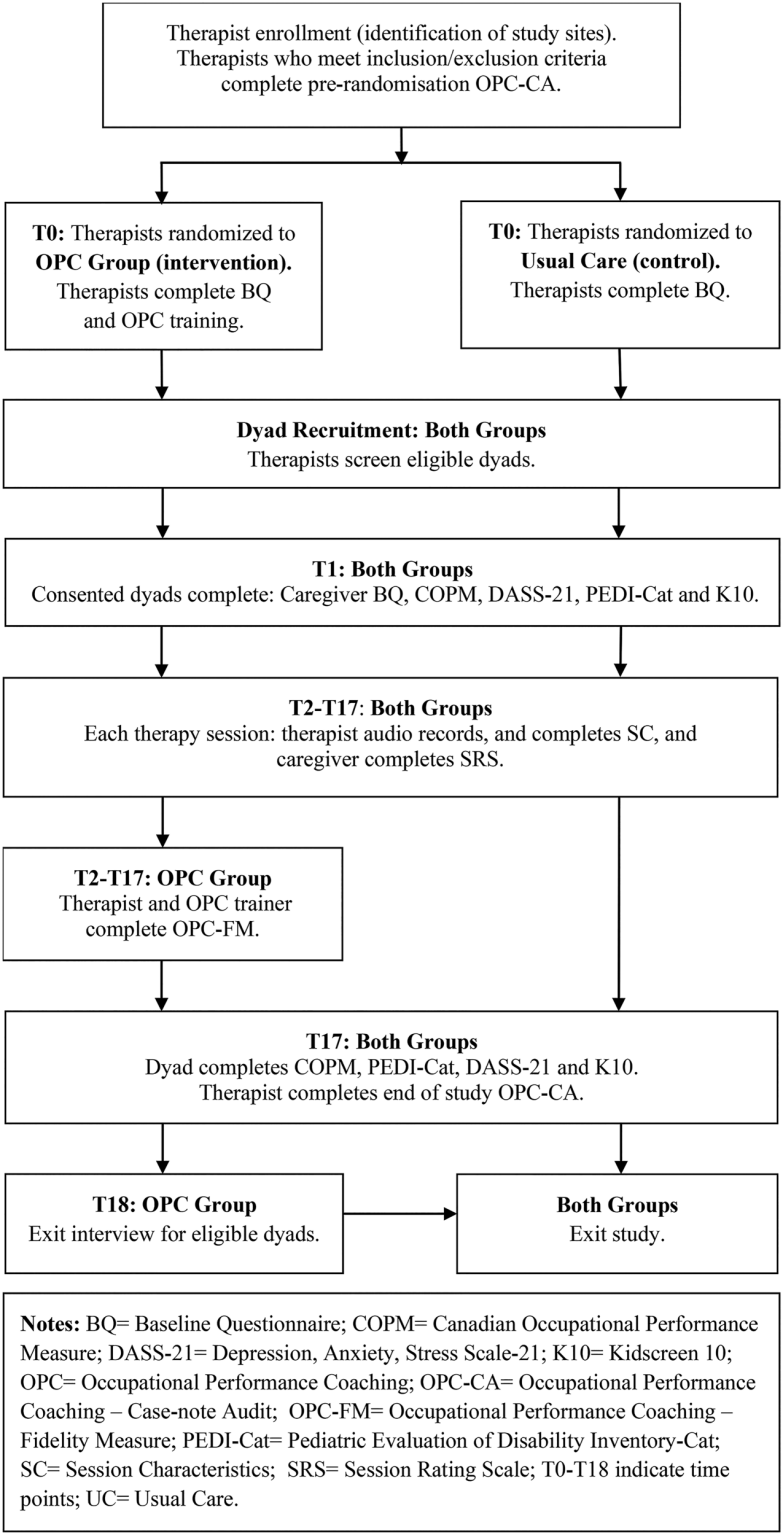
MANA study flowchart. The study population and handling were consistent with the CONsolidated Standards of Reporting Trials (CONSORT).

This trial (entitled the MANA study: Meaning, Agency and Nurturing Autonomy through OPC) will examine if OPC is superior to usual care using a single-blind, 2-arm parallel group, cluster RCT design, with 1:1 allocation. Clustering will take place at the level of the therapist. See Figure 1 for a flowchart of the MANA study and Supplemental Additional File 1 for the full protocol which has been compiled following the SPIRIT (Standard Protocol Items: Recommendations for Interventional Trials) guidelines (Chan et al., 2013).

#### Study Setting and Recruitment

All participants will be recruited from publicly funded Aotearoa/New Zealand community-based rehabilitation services that provide rehabilitation services to children with ND and their families. Each service operates independently, with varying workplace cultures and service processes. Consultations are delivered either in-person or via tele-health.

### Randomization Process

Therapists represent clusters in the study design with study participants defined as caregivers and their children with ND (dyads). After completion of a retrospective case note audit, therapists (N ≥ 14) will be randomized into the OPC or Usual Care groups via a computer-generated randomization sequence, stratified by service (Harris et al., 2009). Thus, services with multiple therapists enlisted in the study will have a mixture of control and intervention group therapists. Therapists in both groups will then each recruit child–caregiver dyads to the study from successive eligible referrals to the service until the total sample size (N = 84) is reached. To enable adequate recruitment of Māori dyads for equal explanatory power for ethnicity, therapists will continue to invite Māori to participate until 42 Māori dyads are recruited (potentially increasing the total sample size to N = 126), or data collection has ended.

### Inclusion and Exclusion Criteria

#### Therapists

Eligible therapists will be registered occupational therapists, physiotherapists, or speech and language therapists, working in publicly funded community rehabilitation services, with a minimum of six months’ experience working with children with ND and their families.

#### Child–Caregiver Dyads

Children and caregivers will be recruited as dyads with the caregiver as the primary intervention recipient. For eligible dyads the caregiver is the primary (or co-primary) caregiver of the child including parents, foster caregivers, whāngai (Māori customary practice when a child is raised by non-birth parents); the child is aged 2–18 (inclusive) with a primary diagnosis of ND, for which the caregiver has sought rehabilitation; the caregiver has one or more goals in relation to themselves, their child or their family, and three or more 1:1 therapy sessions are anticipated by the treating therapist.

The dyad will be ineligible for the study if:

1. the caregiver lacks sufficient English language skills to complete the outcome measures, because the reliability of measures depends on English language skills,
2. the child has been referred solely for adaptive equipment (e.g., wheelchairs), because equipment prescription is outside the scope of this study,
3. the child or caregiver plan to start an alternative rehabilitation intervention during the study period (e.g., Botox injections) to eliminate potential confounding variables of interventions known to have substantial initial impacts on children's functional ability.

### Sample Size

The Minimal Clinically Meaningful Difference (MCID) on the COPM is indicated by a change in occupational performance scores of two points on a 10-point scale (Law et al., 2014). A minimum sample size of 46 dyads is required to detect the MCID with 90% power at a two-sided alpha of 0.05, assuming a population standard deviation in COPM scores of two points (Graham et al., 2013). For an ANCOVA analysis of a cluster RCT, this sample size must be multiplied by a design effect to account for the correlation structure of the data (Rutterford et al., 2015). The final sample size has thus been inflated to 84 dyads (spread evenly between 14 or more therapists) to account for clustering (design effect = 1.2), treatment contamination in the control group (20%), and participants lost-to-follow-up (15%). We seek to recruit a minimum of 42 dyads where the caregiver is of Māori ethnicity, to allow for an adequately powered subgroup analysis with 80% power to detect a difference in treatment effect on the COPM.

For post-intervention interviews, 10 Māori who have received a minimum of one high-fidelity OPC session will be successively invited to participate. This sample size is based on prior research (Walker et al., 2017) and will be adjusted according to information power (Braun & Clarke, 2022; Malterud et al., 2016).

In relation to blinding: First, a research fellow (RF), blinded to group allocation, will collect all outcome measures in both intervention and control groups. The RF will have no contact with therapists or access to study data about therapists. Second, dyads will be blind to group allocation. Therapists will not be blinded to group allocation but will be requested not to refer to their group allocation or OPC by name to caregivers and children. Third, the biostatistician will not be blinded however the statistical analysis plan and statistical analysis code will be prepared a priori to the collection of the 16-week outcome data to ensure that analysis processes are not influenced by the data collected.

## Study Groups

Pre-randomization, a retrospective case note audit will be conducted using the OPC Case-note Audit tool (OPC-CA) (Graham & Ziviani, 2021a) to indicate the extent to which “usual care” for all enrolled therapists aligns with OPC.

### Intervention Group

Therapists randomized to the OPC group will undertake the recommended minimum of 24 hours (Graham & Ziviani, 2021a) of OPC training over four weeks. Training involves the completion of eight two-hour teleconference sessions and an eight-hour self-directed study package. Therapists will be provided with a published OPC manual (Graham et al., 2021), and undertake practice with feedback from the trainer. No modifications to OPC are planned for this study.

#### Occupational Performance Coaching

**Figure 2.**
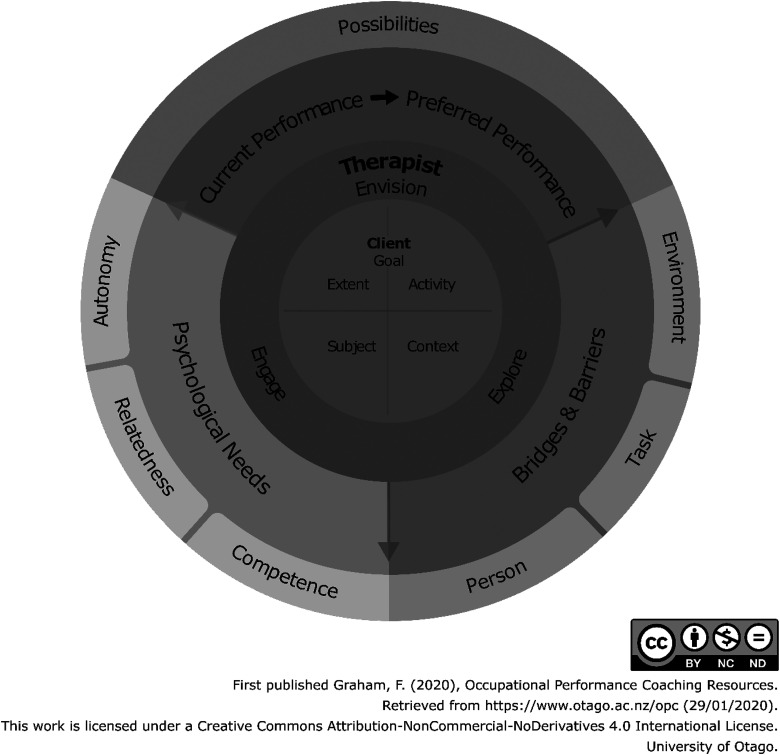
Collaborative Performance Coaching is a unique analytical technique within Occupational Performance Coaching (OPC).

Therapists trained in OPC are taught to commence by asking questions which guide caregivers to identify their preferred future as goal statements. Goals describe participation in life situations (Imms et al., 2016) and occupational performance (Graham & Ziviani, 2021b). Thus goals in OPC specify observable actions in the context of life situations (i.e., home, school, or community), and reflect the personally meaningful change from caregivers’ perspectives. Therapists learn to move conversations in OPC through a process of Collaborative Performance Analysis from extensive *envisioning* of the preferred future-state goal, *exploring* bridges and barriers to goal progress, and *engaging* caregivers through attention to their psychological needs for autonomy, relatedness, and competence (see Figure 2).

Key elements of OPC are listed in the OPC Fidelity Measure (OPC-FM) https://www.otago.ac.nz/opc/resources, designed according to the guidelines of the Treatment Fidelity Group (Bellg et al., 2004).

In implementing OPC, therapists do not use specialized equipment or materials or undertake assessments of impairments. No hands-on or directive (e.g., therapist arranging environment) methods are used with either the caregiver or child unless requested by the caregiver in the context of trialing ideas for collaborative analysis of their usefulness.

#### OPC Dosage

Therapists will engage with caregiver–child dyads in sessions of OPC over a period of up to 16 weeks, whereby a therapy “session” is defined as a therapeutic interaction (i.e., non-administrative) of at least 30 minutes duration. Therapists anticipate three or more sessions for each dyad, and will deliver OPC to the caregiver alone, or to the dyad, either in-person in the home, community, or clinic setting, or via telephone/video conference. OPC sessions typically take 45–60 minutes but can range from 20 to 90 minutes. Data will be collected until either therapy ends, or 16 weeks from the initial therapy session. Fidelity to OPC will be monitored using the OPC-FM and the OPC-CA (Graham & Ziviani, 2021a).

### Usual Care Group

The comparison group for this study will receive “Usual Care,” which is known to be highly variable in content and dose across Aotearoa/New Zealand (Widdowson et al., 2015), and internationally (Anaby et al., 2017). Although Usual Care as a control group is problematic (Levack et al., 2019), it remains the best choice in this translational study in which OPC effectiveness is being examined with minimal interruption to other aspects of service delivery.

Prior evaluations of usual care in community paediatric rehabilitation in NZ (Graham et al., 2020; Widdowson et al., 2015) indicate: Goal setting is documented about half of the time; goals seldom address changes in participation in life situations, but rather children's impairments and activities; methods vary from hands on (child) to hands off (talking-based) methods and from child-directed to caregiver-directed; discussion with caregivers during intervention ranges from collaborative to directive and advice-based; provision of advice appears to be a dominant model of practice. Each of these features are deviations from OPC processes. Several strategies are in place to enable the description of what occurs within Usual Care and monitor contamination with the OPC group, including analysis of casenotes (OPC-CA), audio recording and coding of treatment sessions (using the OPC-FM), and collection of dose, location and delivery format of sessions. In addition, the sample size estimate has been set conservatively given the expected variation within the Usual Care group. Data will be collected from each Usual Care therapy session until 16 weeks from the initial session.

## Measures and Procedures

### Outcome Measures

#### Primary Outcome Measure

The primary outcome is children's participation in personally valued life situations, measured 16 weeks after the first recorded therapy session using the COPM (Law et al., 2014). The mean between-group difference will be calculated by adjusting for values at recruitment (T1, see Figure 1). All caregivers, and children with cognitive and communication skills at or above age 8, will be invited to identify up to five life situations which are important to them, allowing formulation of future-oriented participatory goal statements. Consistent with COPM guidelines, caregivers and children will rate current “performance” and then ‘satisfaction with performance’ on 10-point Likert scales. The “performance” scale is the primary outcome.

The COPM has robust psychometric properties with paediatric ND populations, and is widely considered to be a gold standard measure of individualized performance in areas of personal value (Cusick et al., 2006, 2007; Law et al., 2014) and participation (Sakzewski et al., 2007).

### Secondary Outcome Measures

#### Pediatric Evaluation of Disability Inventory Computer Assisted Technology (PEDI-CAT)

Children's activity and participation will also be measured using an online version of the PEDI-CAT (Speedy version) (Haley et al., 2011). Theoretically, therefore we anticipate that improvement in child-focussed goals on the COPM that were addressed in therapy may be associated with improvement in related PEDI domains, particularly Daily Activities, Mobility, and Social/Cognitive domains (Thompson et al., 2018). The PEDI-Cat uses statistical algorithms to select a minimal set of questions based on the child's age, gender, use of a mobility device, and responses to previous questions. Caregivers will indicate their child's ability in the domains of Daily Activities, Mobility, and Social/Cognitive Skills, and Responsibility on 4-point scale. Scaled scores and normative scores (T-scores and age-percentiles) are generated for each domain.

#### KIDSCREEN10

The child reported quality of life will be measured using the KIDSCREEN10. A summary score reflects the overall perceived quality of life and wellbeing. It is validated for children aged 8–18 years. Psychometric analysis indicates adequate reliability and validity (Ravens-Sieberer et al., 2010).

#### Depression Anxiety Stress Scales (DASS-21)

Caregiver mental health will be measured using the short form of the DASS-21 (Crawford & Henry, 2003). Parental mental health is an important consideration in family centred intervention for children with neurodisabilities. Prior research on OPC indicates that parental psychological state may be both a mechanism (Graham et al., 2010) and an outcome of OPC (Chien et al., 2020; Graham et al., 2013) hence DASS21 will be collected pre and post-intervention. The DASS21 is a self-report questionnaire with 21 items using a 4-point response scale to measure negative emotional states over the prior week. The DASS-21 has been shown to demonstrate acceptable to excellent internal consistency and concurrent validity (Antony et al., 1998), is widely used with caregivers of clinical paediatric populations (Poole et al., 2018; Rayan & Ahmad, 2017) and is sensitive to change following OPC (Graham et al., 2013).

### Mechanisms of Change and Fidelity

#### Session Characteristics

At the end of each therapy session all therapists will record the session format (in person, phone, video-conference), length (minutes), and location (i.e., home, pre/school, community, clinic), and who was present and actively involved (i.e., child, sibling, partner, other adult family, and other professionals named by role—e.g., “social worker”). Non-attendance of scheduled sessions will also be recorded to inform the economic analysis.

#### Session Rating Scale (SRS)

At the end of each therapy session, all caregivers will rate their perceptions of the therapeutic alliance using the SRS (Duncan et al., 2003) in which a 4-item visual analog scale is used to assess client perspectives of respect and understanding of practitioner, the relevance of goals and topics, client–practitioner fit, and overall alliance. The scale has robust psychometric qualities (Campbell & Hemsley, 2009).

#### OPC-FM

The OPC-FM (Graham & Ziviani, 2021a) is a brief measure of fidelity that distinguishes OPC from expert-led and impairment-oriented approaches and indicates the quality of OPC application. Iterations of the OPC-FM were applied to over 50 recorded OPC sessions, with 10 therapists, by six raters. Formal psychometric testing of the OPC-FM is needed and planned as part of secondary analyzes within this study. The OPC-FM measures the occurrence of behaviour and the quality of this behaviour, with 14 items focusing on therapist behaviour and four items focusing on client responses. A score of 80% or higher is believed to represent high fidelity to OPC.

To monitor fidelity, OPC group therapists will self-administer the OPC-FM following the initial session, and one subsequent self-selected session with each dyad. Experienced OPC trainers will also score the OPC-FM based on audio-recordings of sessions. Therapists will be provided individualized feedback on OPC fidelity and offered additional 1:1 training until fidelity reaches adequate levels, but will not be removed from the primary analysis. Separately, raters, blind to group allocation, will complete the OPC-FM on a random sample of audio recordings from both groups to identify contamination between groups and enable unbiased reporting of treatment delivery.

#### OPC-CA

The OPC-CA (Graham & Ziviani, 2021a) is a 13-item measure providing a broad indication of the implementation of OPC, from case-note records. Items capture key elements of OPC including goal-oriented conversation, analysis, action planning, evaluation, and generalization. Relational elements of OPC, such as the use of empathy to engender client trust, are not included given these are unlikely to be documented.

The OPC-CA will be conducted on two occasions: first, prior to therapist randomization, with a retrospective sample of three case note entries for each of three dyads (closed cases), who meet the study criteria; second, at T16 with three case note entries selected for each dyad who participated in the study. For the OPC group, this will allow monitoring of adherence to OPC; for the Usual Care group, this will help to determine the continuity of usual care and any contamination of the Usual Care group.

### Qualitative Measure

A post-intervention semi-structured interview will explore Māori caregiver experiences (*n *= 10) of engaging in OPC, in the context of their cultural expectations of health professionals. Interview questions will be guided Kaupapa Māori principles (Smith, 2013). Interviews will be conducted by a Māori research fellow with adherence to culturally informed processes of engagement (Pitama et al., 2017).

## Statistical Methods

Participant data will be analyzed as-randomized (intention-to-treat, ITT). Two-sided *P* values will be reported and an alpha of 0.05 used for the construction of confidence intervals and for determining statistical significance. The analysis will be performed using R specialist statistical software (R Core Team, 2021). A detailed statistical analysis plan (SAP) will be prepared by the study biostatistician and agreed upon by the PI prior to any analysis of the data.

Differences between groups in outcome measures (COPM scores, DASS-21, and PEDI-CAT scores at 16 weeks) will be estimated with 95% confidence intervals using a “constrained baseline analysis” where a linear mixed-effects model is fitted to the individual level data with time and treatment included as fixed effects. Correlations within the data will be accounted for by incorporating the following random effects; cluster, an individual within cluster, and time within cluster, and a Kenward–Roger correction will be applied due to the small number of clusters (Hooper et al., 2018). Psychometric analysis of therapist self-completed and researcher-completed OPC-FM scores will be conducted to assess the validity and reliability of this instrument. Researcher completed OPC-FM scores, Session Characteristics, and SRSs will be summarised first by a therapist, and then by the treatment group. Linear regression analysis adjusting for clustering will be used to estimate mean treatment group differences with 95% confidence intervals.

### Additional Analyzes

Baseline therapist and dyad characteristics will be summarised for the whole ITT population using means (standard deviations), medians (interquartile ranges), and frequencies (percentages) as appropriate. Summaries will be tabulated by the treatment group but no hypothesis testing will be undertaken. Goals documented by therapists and identified in the OPC-CA for both intervention and control groups will be compared against goal criteria for OPC and reported descriptively (i.e., counts, percent) (Graham & Ziviani, 2021a).

Adjusted analysis of primary and secondary outcome measures (COPM, PEDI-cat, and DASS-21) will be undertaken by adjusting for the following baseline variables as fixed effects in the model; therapist profession (OT, PT, and SLT), therapist years of experience, caregiver–child socio-economic status, caregiver ethnicity (Māori vs. non-Māori), caregiver education level, caregiver DASS-21, child age, and child ND.

A sensitivity analysis will be conducted on the primary outcome using the as-treated population. Caregiver–child dyads will have been deemed to have received the intervention (OPC) if more than half (two out of the three) OPC-FM assessments (researcher-completed or bias-adjusted therapist self-completed) for the caregiver–child dyad score 80% or above.

The heterogeneity of treatment effects will be assessed using the framework presented by Kent et al., 2010. An outcome prediction score will be calculated for each participant using a multivariable linear regression model including selected baseline variables (excluding the treatment group). A second linear regression model will be fitted including the outcome prediction scores, treatment group, and interaction between the two as independent variables, and observed outcome scores as the dependent variable. In addition, subgroup analysis of Māori caregiver–child dyads is planned a priori. Where interaction effects are found, treatment effects with 95% CI will be presented by each subgroup in tabular form, with continuous predictors dichotomized into approximately equal-sized groups.

Interview data with Māori caregivers will be analyzed by senior Māori researchers using reflexive thematic analysis (Braun & Clarke, 2022). This analysis will be underpinned by Kaupapa Māori Research principles (Smith, 2013) that privilege the perspectives of Māori participants and ensure the interpretation of data is culturally appropriate (Jones et al., 2010).

#### Economic Analysis

Cost-effectiveness analysis (Drummond, 2015) from a health service perspective will be employed. As the intervention is planned to be delivered within a 12-month timeframe, discounting will not be used. Sensitivity analysis will investigate the major potential factors that may influence cost-effectiveness including variation in intervention effectiveness, adherence to OPC, dosage associated with effectiveness, and variation in intervention costs, particularly staff input and travel costs. Health service costs will include therapist training, intervention, and travel time and will exclude lost productivity and time costs of family members. Health effectiveness will be based on COPM (MCID ≥ 2 points). As therapists will be recruited throughout the North and South Islands, the study design is expected to provide information regarding national transferability.

### Analysis Population

All dyads who formally consent to participate, and complete at least one intervention session, will be included in the analysis as the “as randomized population.” This population is the primary analysis population for all analyzes.

A subset of the population who meet all inclusion and exclusion criteria and have completed one or more sessions with a therapist enrolled in the study will be included in the per-protocol population. All decisions on participant exclusions from the per-protocol population will be made prior to database closure and will be justified in the clinical study report.

### Missing Data

The presence of missing data in this cluster RCT will be handled as recommended by Fiero et al. (2016). Firstly, all efforts will be made to optimize collection of the primary measure (COPM). Secondly, a likelihood-based mixed model including all collected data will be used for the primary analysis. Mixed models provide the greatest power and are valid for cases where data is missing at random (MAR). Finally, a sensitivity analysis will be performed where subjects’ missing outcome data will be assumed to have not improved at all during the study.

## Discussion

This RCT protocol outlines a significant step on the continuum of translational research (Vukotich, 2016), building on previous efficacy and feasibility studies to evaluate its effectiveness in the service delivery contexts it is intended for. Studies embedded in real-world contexts are needed to understand and address the factors that contribute to the science-implementation gap (Olswang & Prelock, 2015) for OPC. Subsequent studies examining the scalability of OPC implementation can then be planned. Distinctively, this study incorporates multiple strategies to report and monitor intervention fidelity and the nature of usual care as a comparison group. This study provides transparency to usual care in relation to therapist inputs, health outcomes, and economic impacts. This study also illustrates translational research for complex interventions (Murray et al., 2010) situated in the occupational therapy and rehabilitation fields.

With the recruitment of the intended sample size, important secondary analyzes will be possible exploring the effectiveness of OPC for Māori and how OPC was experienced by Māori caregivers. Internationally, these analyzes with Māori extend our understanding of the relationship between culture and responsive occupational therapy interventions.

### Limitations

This study may be limited by recruitment of the target sample size of child–caregiver dyads given the Aotearoa/New Zealand population size and the ongoing effects of COVID-19 on participating services. COVID-19 may also affect service delivery patterns in a range of ways that do not provide an accurate representation of the usual service delivery context, however, this will be balanced for both OPC and Usual Care groups. The known high variability of usual care as a comparison may reduce the certainty of findings and challenge the pooling of data in future meta-analyzes of OPC research. The established known therapeutic effects of goal setting (Graham et al., 2013; Locke & Latham, 2002) are an unavoidable limitation of using goal setting and individualized goal progress as the primary outcome. This may have the effect of diminishing the change observed in the OPC group relative to the Usual Care group, producing a conservative assessment of the treatment effect. Therapists from the same agencies may be randomized to either group, introducing a risk of contamination. This was a necessary risk in order to achieve the study sample size, and strategies were put in place to minimize contamination within sites. Contamination will be identified through analysis of the retrospective case note audit and OPC-FM findings.

## Conclusions

The proposed study will determine the effectiveness of OPC to improve the occupational performance and participation of children with ND and their families when implemented in existing service delivery contexts. Findings will inform future strategies to reduce the science-implementation gap for OPC.

## Key Messages

- OPC is a transdisciplinary intervention conceptually grounded in occupational therapy theory, and coaching-related principles of autonomy, self-determination, and behaviour change.
- OPC has evidence of effect under controlled conditions but translational research studies, situated in service delivery contexts, are now needed.
- This study protocol enhances the scholarship of occupational therapy research through providing transparency of a planned RCT of the effectiveness of OPC.

## Supplemental Material

sj-pdf-1-cjo-10.1177_00084174231160976 - Supplemental material for Occupational Performance Coaching for Children With Neurodisability: A Randomized Controlled Trial Protocol Supplemental material, sj-pdf-1-cjo-10.1177_00084174231160976 for Occupational Performance Coaching for Children With Neurodisability: A Randomized Controlled Trial Protocol by Fiona P. Graham, Jonathan A. Williman, Laura N. Desha, Deborah Snell, Bernadette Jones, Tristram R. Ingham, Anna Latu, Jasjot K. Maggo, Annemarei Ranta, and Jenny Ziviani in Canadian Journal of Occupational Therapy

## Notes

1. The more inclusive term of ‘caregiver’ is used from this point to reflect the diverse social and biological roles that primary caregivers may have.
