# Supplementary material for: Occupational Performance Coaching for Children With Neurodisability: A Randomized Controlled Trial Protocol
Source: Can J Occup Ther. 2023 Mar 15;91(1):4–16. doi: 10.1177/00084174231160976 (PMC10903119; doi:10.1177/00084174231160976)
Supplement: sj-pdf-1-cjo-10.1177_00084174231160976 - Supplemental material for Occupational Performance Coaching for Children With Neurodisability: A Randomized Controlled Trial Protocol [file sj-pdf-1-cjo-10.1177_00084174231160976.pdf]

Health and Disability Ethics Committees

Health and Disability Ethics Committees
Ministry of Health
133 Molesworth Street
PO Box 5013
Wellington
6011

hdecs@health.govt.nz

20 August 2020

Dr Fiona Graham
University of Otago
Christchurch
University of Otago
Christchurch 8013

Dear Dr Graham

Re:	**Ethics ref:**	**20/STH/93**
	Study title:	A single blind, 2-arm parallel group cluster randomized controlled trial to study the effect on social participation, health and wellbeing of Occupational Performance Coaching compared to usual care for children with neuro-developmental disability and their caregivers.

I am pleased to advise that this application has been *approved* by the Southern Health and Disability Ethics Committee. This decision was made through the HDEC-Full Review pathway.

Summary of outstanding ethical issues

The main ethical issues considered by the Committee which require addressing by the Researcher are as follows.

1. Cover letter point 5: Please note that information must be kept for 10 years after the youngest participant reaches the age of 16 NOT retained until they reach 16 years as stated in the letter and in section 8.2.4 of the protocol.

Conditions of HDEC approval

HDEC approval for this study is subject to the following conditions being met prior to the commencement of the study in New Zealand. It is your responsibility, and that of the study's sponsor, to ensure that these conditions are met. No further review by the Southern Health and Disability Ethics Committee is required.

Standard conditions:

1. Before the study commences at *any* locality in New Zealand, all relevant regulatory approvals must be obtained.

2. Before the study commences at *any* locality in New Zealand, it must be registered in a clinical trials registry. This should be a WHO-approved registry (such as the Australia New Zealand Clinical Trials Registry, www.anzctr.org.au) or https://clinicaltrials.gov/.

3. Before the study commences at *each given* locality in New Zealand, it must be authorised by that locality in Online Forms. Locality authorisation confirms that

the locality is suitable for the safe and effective conduct of the study, and that local research governance issues have been addressed.

<u>After HDEC review</u>

Please refer to the *Standard Operating Procedures for Health and Disability Ethics Committees* (available on www.ethics.health.govt.nz) for HDEC requirements relating to amendments and other post-approval processes.

Your next progress report is due by 20 August 2021.

<u>Participant access to ACC</u>

The Southern Health and Disability Ethics Committee is satisfied that your study is not a clinical trial that is to be conducted principally for the benefit of the manufacturer or distributor of the medicine or item being trialled. Participants injured as a result of treatment received as part of your study may therefore be eligible for publicly-funded compensation through the Accident Compensation Corporation (ACC).

Please don't hesitate to contact the HDEC secretariat for further information. We wish you all the best for your study.

Yours sincerely,

Mrs Helen Walker
Acting Chairperson
Southern Health and Disability Ethics Committee

Encl: appendix A: documents submitted
appendix B: statement of compliance and list of members

Appendix A
Documents submitted

Document	*Version*	*Date*
CV for CI	1	27 May 2020
CVs for other Investigators	1	27 May 2020
CVs for other Investigators	1	27 May 2020
CVs for other Investigators	1	27 May 2020
CVs for other Investigators	1	27 May 2020
CVs for other Investigators	1	27 May 2020
CVs for other Investigators	1	27 May 2020
Evidence of scientific review	1	27 May 2020
Survey/questionnaire	1	27 May 2020
Investigator's Brochure	1	27 May 2020
PIS/CF: Combined file of Therapist, Caregiver and child 16-18 years consent and PIS.	2	27 May 2020
PIS/CF for persons interested in welfare of non-consenting participant: Assents for children 5-13 and 14-15 years.	2	27 May 2020
Protocol	3	27 May 2020
Evidence of sponsor insurance	1	27 May 2020
Application		27 May 2020
Covering Letter: Cover letter amendments with Poulsen correspondence attached.	2	06 August 2020
PIS/CF for persons interested in welfare of non-consenting participant: Amended PIS and CF for caregivers WITH track changes	2	05 August 2020
PIS/CF for persons interested in welfare of non-consenting participant: Amended PIS and CF for caregivers WITHOUT track changes	2	05 August 2020
PIS/CF: Amended PIS and CF for caregivers WITH track changes	2	05 August 2020
PIS/CF: Amended PIS and CF for caregivers WITHOUT track changes	2	05 August 2020
PIS/CF: Amended PIS and CF for therapists WITH track changes	2	05 August 2020
PIS/CF: Amended PIS and CF for therapists WITHOUT track changes	2	05 August 2020
PIS/CF: Amended PIS and CF for child/young people WITH track changes	2	05 August 2020
PIS/CF: Amended PIS and CF for children/young people WITHOUT track changes	2	05 August 2020
Protocol: Amended protocol WITH track changes	2	05 August 2020
Protocol: Amended protocol WITHOUT changes	2	05 August 2020
Survey/questionnaire: This is the same document that was inadvertently uploaded as 'declined letter' doc type on 5/8/2020. Please delete 'declined letter' doc version.	3	06 August 2020
Evidence of scientific review: Three full peer reviews from HRC.	2	06 August 2020
Response to Request for Further Information		

Appendix B
Statement of compliance and list of members

Statement of compliance

The Southern Health and Disability Ethics Committee:

- is constituted in accordance with its Terms of Reference
- operates in accordance with the *Standard Operating Procedures for Health and Disability Ethics Committees*, and with the principles of international good clinical practice (GCP)
- is approved by the Health Research Council of New Zealand's Ethics Committee for the purposes of section 25(1)(c) of the Health Research Council Act 1990
- is registered (number 00008713) with the US Department of Health and Human Services' Office for Human Research Protection (OHRP).

List of members

Name	*Category*	*Appointed*	*Term Expires*
Dr Sarah Gunningham	Lay (other)	05/07/2019	05/07/2022
Dr Pauline Boyles	Lay (consumer/community perspectives)	05/07/2019	05/07/2022
Dr Paul Chin	Non-lay (intervention studies)	27/10/2018	27/10/2021
Dr Devonie Eglinton	Non-lay (intervention studies)	18/07/2016	18/07/2019
Mr Dominic Fitchett	Lay (the law)	05/07/2019	05/07/2022
Assc Prof Mira Harrison-Woolrych	Non-lay (intervention studies)	28/06/2019	28/06/2020
Professor Jean Hay-Smith	Non-lay (health/disability service provision)	31/10/2018	31/10/2021

Unless members resign, vacate or are removed from their office, every member of HDEC shall continue in office until their successor comes into office (HDEC Terms of Reference)

http://www.ethics.health.govt.nz
